# Flame-Retarding Properties of Injected and 3D-Printed Intumescent Bio-Based PLA Composites: The Influence of Brønsted and Lewis Acidity of Montmorillonite

**DOI:** 10.3390/polym14091702

**Published:** 2022-04-21

**Authors:** Raíssa Carvalho Martins, Simone Pereira da Silva Ribeiro, Michelle Jakeline Cunha Rezende, Regina Sandra Veiga Nascimento, Marco Antonio Chaer Nascimento, Marcos Batistella, José-Marie Lopez-Cuesta

**Affiliations:** 1 Instituto de Química, Universidade Federal do Rio de Janeiro, Cidade Universitária, CT, Bloco A, Rio de Janeiro 21941-909, RJ, Brazil; spsilva@iq.ufrj.br (S.P.S.R.); michelle@iq.ufrj.br (M.J.C.R.); rsandra@iq.ufrj.br (R.S.V.N.); chaer@iq.ufrj.br (M.A.C.N.); 2 Polymères Composites et Hybrides (PCH), IMT Mines Alès, 6, Avenue de Clavières, 30319 Alès, France; marcos.batistella@mines-ales.fr (M.B.); jose-marie.lopez-cuesta@mines-ales.fr (J.-M.L.-C.)

**Keywords:** intumescent biocomposite, PLA, lignin, flame/fire retardancy, 3-D printing, additive manufacturing, montmorillonite acidity

## Abstract

The influence of processing intumescent bio-based poly(lactic acid) (PLA) composites by injection and fused filament fabrication (FFF) was evaluated. A raw (ANa) and two acidic-activated (AH2 and AH5) montmorillonites were added to the intumescent formulation, composed by lignin and ammonium polyphosphate, in order to evaluate the influence of the strength and the nature (Brønsted or Lewis) of their acidic sites on the fire behavior of the composites. The thermal stability and the volatile thermal degradation products of the composites were assessed. The injected and 3D-printed composites were submitted to cone calorimeter (CC), limit oxygen index (LOI), and UL-94 flammability tests. A similar tendency was observed for the injected and 3D-printed samples. The high density of strong Lewis sites in AH2 showed to be detrimental to the fire-retarding properties. For the CC test, the addition of the intumescent composite reduced the peak of heat released (pHRR) in approximately 49% when compared to neat PLA, while the composites containing ANa and AH5 presented a reduction of at least 54%. However, the addition of AH2 caused a pHRR reduction of around 47%, close to the one of the composite without clay (49%). In the LOI tests, the composites containing ANa and AH5 achieved the best results: 39% and 35%, respectively, for the injected samples, and 35 and 38% for the 3D-printed samples. For the composite containing AH2 the LOI values were 34% and 32% for injected and 3D-printed samples, respectively. Overall, the best performance in the flammability tests was achieved by the composites containing clays with only weak and moderate strength acidic sites (ANa and AH5).

## 1. Introduction

Additive manufacturing, most commonly known as three-dimensional (3D) printing, is a growing technique for the development of advanced materials, especially for polymers, due to their easy processability, versatility of applications (such as in health and aerospace domains), and possibility to manufacture complex-shaped materials with a low cost [1]. Among the various techniques of additive manufacturing, the fused filament fabrication (FFF), usually applied for low-temperatures polymers [1], is well-known due to its simplicity, high-speed, and low-cost. The FFF technique is able to create 3D objects through deposition, layer by layer, of a molten material filament through movements in the X-Y plane over a movable plate. As soon as the first layer deposition is finished, the plate moves downward (in the *Z* axis) and a new layer is added [2]. This process is repeated until the completion of the designed object.

A recent and challenging domain of application of the additive manufacturing is in the production of flame-retarding materials, enabling the conception of advanced, new-designed, and low-cost products [3]. Some polymeric matrix, such as poly(amide) 6 (PA6), poly(lactic acid) (PLA), and acrylonitrile butadiene styrene (ABS) have already been tested for the production of fire-retarding composites through the FFF technique [4]. Despite the increasing economic importance of the additive manufacturing in the worldwide market [5] and the growing interest in using bio-based materials, only a few works developed 3D-printed intumescent flame-retarding PLA composites by FFF [1]. Intumescent PLA nanocomposites with melamine polyphosphate and Cloisite 30B were produced by FFF and hot press molding [6]. Different formulations of flame-retarded PLA containing ammonium polyphosphate, melamine cyanurate, and phyllosilicates (Cloisite 30-B and sepiolite) were studied, processed by FFF and injection [7]. A flame-retardant PLA composite was developed by FFF and hot press molding [8] with the addition of ammonium polyphosphate and resorcinol bis(diphenyl phosphate).

The production of an intumescent flame-retardant composite consists of adding an intumescent formulation, composed by an acid source, a blowing agent, and a carbonific agent, to a polymeric matrix during the processing step. A conventional system [9,10] consists of pentaerythritol (PER) as the polyhydroxylated carbonaceous compound and ammonium polyphosphate (APP) as both the blowing agent and acid source. There are two possible mechanisms proposed for the initial reactions which would take place as the temperature increases up to 280 °C: according to the first [11] one, a direct reaction occurs between APP and PER, producing phosphate esters, while the second one [10] considers that, instead of a direct reaction, the APP decomposes producing acidic species such as pyrophosphates and phosphoric acid, which would then react with the PER, giving rise to phosphate esters. Over 280 °C and under 350 °C, the degradation of the phosphate esters produces unsaturated species, which then undergo Diels–Alder reactions [12], leading to a layered structure, called char, composed of condensed aromatic rings bounded to each other by phosphates species [10]. In that temperature range, the volatile products originated from the APP decomposition causes the swelling of the char, creating a rigid, intumescent layer that acts as a barrier between the material and the external environment, inhibiting the exchange of the combustible volatile products (fuel), heat (thermal energy), and oxygen (oxidizing agent), ceasing the flame.

Many works seek to develop more efficient systems by adding aluminosilicate synergistic agents, such as zeolites [13,14,15] and clays [16,17,18,19,20], to the intumescent formulations. Overall, the authors address the synergic effects observed in the formation of aluminophosphate species [21,22,23] that would connect the layers of condensed aromatic rings, enhancing the char flexibility during the swelling process, consequently minimizing the generation of cracks and holes, which are major drawbacks for the efficiency of the intumescent layer at higher temperatures [10,16]. Moreover, the addition of mineral fillers reduces the total amount of combustible material and the rate of diffusion of oxygen into the polymer, by forming an inorganic barrier on the surface of the burning polymer, protecting it from the external heat source [24].

Nevertheless, only few works investigated the influence of the acidity of aluminosilicates on the flame-retarding properties of intumescent composites [23,25,26,27,28,29]. Montmorillonites are well-known catalysts for esterification reactions in organic liquid media and many works try to correlate a better catalytic performance to the presence of Brønsted acidic sites [30,31,32,33]. Furthermore, montmorillonite (Mt) presents some favorable features: it exhibits swelling properties, cation exchange capacity [34], and its surface acidity can be modified through an acidic activation [35]. Methyl esterification reactions between methanol and carboxylic acids (acetic and lauric acids) were performed, catalyzed by acid-activated smectites with a high content of Mt [33]. It was found that the higher the ratio of Brønsted/Lewis acidic sites, the higher the esterification conversion achieved. The authors attribute this result to the smaller size of the proton (Brønsted acid sites) compared to the Lewis acid sites, which facilitates the protonation of the oxygen of the carbonyl group of the carboxylic acid, favoring the nucleophilic attack by methanol. A pioneer work [29] revealed the influence of the strength and the nature of the montmorillonite acid sites on the flame-retarding properties of a composite made of poly(propylene), APP, PER, and acid-activated Mt. A better performance in the flammability tests was achieved by the composites containing montmorillonites with an excess of Brønsted sites relative to the Lewis ones, and preferentially with moderate-strength Brønsted sites relative to the strong ones. The presence of the Brønsted acidic sites is fundamental until 280 °C, when esterification reactions between APP and PER start to occur. The conclusions of [33] corroborates the results obtained in our previous study [29].

The current society demand for more sustainable materials inspirited the search for bio-based and biodegradable materials in order to replace the commonly used originated from the oil industry. Some works seek to replace the components of the intumescent formulation by more sustainable ones, such as starch [36], tannic acid [37,38], chitosan [39], and lignin [40,41]. Among them, lignin presents particular interest for being a residue from the paper industry [42], as well as for its good thermal stability and aromatic chemical structure, resulting in a high ability for char formation [41]. Lignin is already used in many intumescent formulations [36,40,43,44] and seems to be a potential candidate to replace pentaerythritol, due to its highly cross-linked polyphenolic structure, with abundant aliphatic and aromatic carboxylic groups [36,43]. Moreover, many efforts have been made in the intumescent polymeric composites domain in order to replace the olefin polymers by bio-based ones, such as poly(3-hydroxybutyrate) (PHB) [45], polybutylene succinate (PBS) [41], and poly(lactic acid) (PLA) [46,47,48,49,50]. Amidst the bio-based polymers, PLA presents some competitive advantage such as: a low emission of greenhouse gases during degradation, less energy is required for its production [51], it is compostable [52], it comes from renewable sources, it is a high-strength and high-modulus thermoplastic, and it is easily processable [51].

This work aims to investigate the influence of Brønsted and Lewis acidity of acidic-activated montmorillonites on the flame-retardancy and mechanical properties of intumescent bio-based composites produced by both injection molding and FFF techniques. Ammonium polyphosphate, lignin, raw and acidic-activated montmorillonites were employed as the intumescent formulation in a PLA matrix. To the best of our knowledge, this is the first time that a study of the influence of the montmorillonite acidity is conducted in an intumescent bio-based PLA composite with high processability either for additive manufacturing or injection molding. With the comprehension of the role played by the montmorillonites acidic sites on the reactions involved in the intumescence, and how it affects the flame-retarding properties, it is possible to open-up the way for the development of higher-performant and safer intumescent composites able to be submitted to additive manufacturing, in addition to the traditional processing techniques.

## 2. Materials and Methods

### 2.1. Materials

The intumescent formulation consisted of ammonium polyphosphate (APP), purchased from Clariant under the trade name Exolit AP 422, and alkaline lignin (Lig), supplied by Tokyo Chemical Industry (Tokyo, Japan) (product code L0082). Polylactic acid (PLA), supplied by NatureWorks (Minnetonka, MN, USA) under the trade name Ingeo™ Biopolymer 2003D, was used as the polymeric matrix. Raw, sodic montmorillonite (ANa) originated from Paraíba, Brazil, was submitted to an acidic activation with 4 mol L^−1^ H_2_SO_4_ solution, for 2 and 5 h, at a concentration of 10% m/v mineral clay, to promote an increase in the concentration of the acidic sites, as described in a previous work [29]. The acidified clays were labelled as AH2 (for 2 h treatment) and AH5 (for 5 h treatment).

### 2.2. Montmorillonite Characterization

The characterization of the mineral clays was reported in a previous work [29]. Table 1 presents the concentration of Brønsted (Bpy) and Lewis (Lpy) acidic sites, for ANa, AH2, and AH5, measured by Fourier transform infrared spectroscopy with pyridine adsorption (FTIR-Pyr) in three desorption temperatures (150, 250, and 350 °C). The strength of the acidic sites is associated to the temperature at which pyridine is desorbed. A higher temperature means that more thermal energy is necessary to desorb pyridine from a certain site. Thus, the higher the desorption temperature, the stronger is the site. The acidic sites are classified as “weak” (acidic sites able to retain pyridine only at T ≤ 150 °C), “moderate” (for T ≤ 250 °C), and “strong” (for T ≤ 350 °C). Further details and the references related to the FTIR-Pyr can be found in [29].

### 2.3. Processing of the Composites

#### 2.3.1. Extrusion

In order to produce the polymeric composites without clay, the following concentrations were used (in% m/m): 80.0% of PLA, 17.0% of APP, and 3.0% of Lig; while for the composites containing clays (ANa, AH2 and AH5), the concentrations (in% m/m) were: 78.8% of PLA, 17.0% of APP, 3.0% of Lig, and 1.2% clay. The APP/Lig ratio and the sum of APP and Lig contents were kept constant. The flame retardant loading was based upon [40], in which a synergistic effect was observed in the fire performance of the composites with a ratio of AP/lignin ≥ 3 in the intumescent formulation. In that work, the best flame-retardant performance was obtained for the composite containing 80% PLA + 17% APP+ 3% lignin. A twin-screw extruder (BC21 Clextral 900 mm) with twelve heating zones (T_zone 1_ = 50 °C, T_zones 2,3_ = 195 °C, T_zone 4_ = 190 °C, T_zones 5–12_ = 180 °C), flow rate of 4 kg h^−1^, and a screw rotation of 200 rpm, was employed in order to process the composites.

#### 2.3.2. Injection Molding

A fraction of the pelletized materials (Scheer pelletizer) was submitted to a 50 ton Krauss Maffei (Munich, Germany) injection molding press with mold and screw temperatures of 30 and 180 °C, respectively, in order to produce 100 × 100 × 4 mm^3^ plates. Another fraction of the pellets was submitted to hot pressing in a mini Zamak Mercator press in order to manufacture the dumb-bell-shaped specimens (ISO 1BA) for the tensile tests ISO 1BA (according to ISO 527). The parameters used were: oven temperature of 200 °C during 200 s, mold temperature of 54 °C, pressure of 5 bars, and injection time of 10 s. The raw components and the pellets were dried overnight at 60 °C in compressed air Piovan dryers before each processing step.

#### 2.3.3. Fused Filament Fabrication (FFF)

Calibrated filaments of 2.85 mm for FFF were prepared using a 3devo (Utrecht, The Netherlands) single-screw extruder. Next, square specimens for the Underwriters Laboratory’s UL-94 test (vertical burning test), cone calorimeter, and limit oxygen index (LOI) tests were prepared using an A4v3 FFF equipment from 3NTR (Italy) equipped with a 0.4 mm nozzle. Alternated infill patterns of + 45° and −45° between every layer were also used. The samples made by FFF are identified by the label “3D” at the end of their names. The PLA grade used (Ingeo™ Biopolymer 2003D) did not allow for the production of a flexible neat PLA filament able to be spooled, impairing the feeding of the FFF printer. Consequently, it was not possible to print specimens using the neat PLA. However, the PLA composites were easily printable.

### 2.4. Polymer Composites Thermogravimetric Analysis Coupled to Gas Phase Fourier Transform Infrared Spectroscopy (TGA-FTIR)

Thermogravimetric analysis (TGA) measurements of the extruded pellets were carried out using a SETSYS evolution equipment (Setaram, France), equipped with alumina microbalance pans, under a 10 °C min^−1^ heating rate, a 100 cm^3^ min^−1^ synthetic air flow, and a temperature range from 30 to 900 °C, coupled to a gas phase IS10 Thermo IR spectrometer (temperature of transfer line of 200 °C and time interval between measurements of 0.4 min). The experimental and theoretical TGA curves of the composites were compared in order to evaluate the interaction between the components. The theoretical curves were calculated considering the individual contribution of each additive in its respective concentration to the composite’s TGA, as shown in Table 2. In order to evaluate the effect of just the clay addition, eliminating the influence of the possible reactions between APP and lignin, the theoretical mass loss of the intumescent formulation (*M*_IF_) was calculated based on the experimental TGA curve of the mixture of APP and lignin at the same proportion used for each composite, as described in Section 2.3.1.

### 2.5. Polymer Composites Scanning Electron Microscopy (SEM)

A Quanta FEG 200 (FEI Company (Hillsboro, OR, USA)) SEM equipment, operating at 10 kV with a back scattered electrons (BSE) detector and under high vacuum was employed to analyze the inner surface of the cryogenic fractured composites (before burning) of the injected samples. The analyzed surfaces were coated with a 20 nm carbon layer.

### 2.6. Flammability Tests

#### 2.6.1. Cone Calorimetry (CC)

Three specimens (100 × 100 × 4 mm^3^ plates) of each injected and 3D-printed samples were submitted to cone calorimetry in a Fire Testing Technology (FTT) equipment, under the following experimental conditions: 24 L s^−1^ air flow, 50 kW m^−2^ external heat flux, a distance between the cone and the sample of 25 mm. The most representative curve for each set of samples was plotted.

#### 2.6.2. Underwriters UL-94 Test

The Underwriters Laboratory’s UL-94 test (vertical burning test; ASTM D 380) enables the assessment of the self-extinguishing time of materials. In this test, a methane burner is placed directly below a 4 mm-thickness specimen clamped to a stand in the vertical position. Some parameters are considered to the classification of the material, such as the occurrence of burning drips and the burnt extension of the specimen, the amount of time during which the material continues to burn after removing the flame, the first (t_1_) and the second (t_2_) flame approximation and the incandescence time (t_3_). The classifications are labelled as V0, the best, when the material is able to quickly extinguish the flame and presents a weak afterglow, without burning drips: (t_1_ or t_2_ for each individual specimen < 10 s and [t_1_ + t_2_] < 50 s for the five specimens); V1, when the material extinguishes the fire in a longer time without burning drips (t_1_ or t_2_ for each individual specimen < 30 s and [t_1_ + t_2_] < 250s for the five specimens) and V2, the lowest ranking, when burning drips are observed, or when the material takes longer to extinguish the flame with burning drips (t_1_ or t_2_ for each individual specimen < 30 s and [t_1_ + t_2_] < 250 s for the five specimens). This test was performed for the injected and 3D-printed samples.

#### 2.6.3. Limiting Oxygen Index (LOI)

The LOI test enables the determination of the minimum oxygen concentration required for supporting the flame combustion of a material; the higher the % O_2_ achieved, the better is its performance. To conduct this test (LOI, ISO 4589-2), a propane flame is approached to the top of a 100 mm × 10 mm × 4 mm specimen placed in the upright position in a controlled atmosphere composed of nitrogen and oxygen. The specimen should extinguish the flame in less than 180 s keeping at least 50% of its length not consumed by the flame. The oxygen concentration is increased gradually and the test is finished when the specimen is no longer able to achieve those requirements. This test was performed in an apparatus composed by a gas flow regulator and a burning chamber with controlled atmosphere for the injected and 3D-printed samples.

### 2.7. Fourier Transform Infrared Spectroscopy (FTIR) of the Char

The FTIR spectra of the chars from the injected samples obtained at the end of the cone calorimeter test were collected on KBr pellets with a sample concentration of 1% in a Vertex 70 FT MIR spectrometer (Bruker (Billerica, MA, USA)) with 4 cm^−1^ resolution and spectral range from 4000 cm^−1^ to 400 cm^−1^.

### 2.8. Melt Flow Rate (MFR)

The MFR of the extruded pellets of PLA and the composites was assessed by a MF20 CEAST Melt Flow Tester (Instron, Norwood, MA, USA), according to ASTM D1238, method A, at 210 °C, 2.16 kg weight. The pellets were dried overnight at 60 °C in a Memmert D06836 vacuum oven before the test.

### 2.9. Tensile Test

Tensile tests of the injected and 3D-printed samples were carried out in a Zwick Roell BZ2 device equipped with 10 N load cell on dumbbell-shaped specimens (geometry type ISO1BA, ISO 527). The Young’s modulus was determined at a cross-head speed of 1 mm min^−1^ through an extensometer. The tensile strength, the yield stress, and the elongation at break were determined at a cross-speed of 50 mm min^−1^. Five specimens of each sample were tested.

## 3. Results and Discussion

### 3.1. Thermal Behaviour

**Figure 1:**
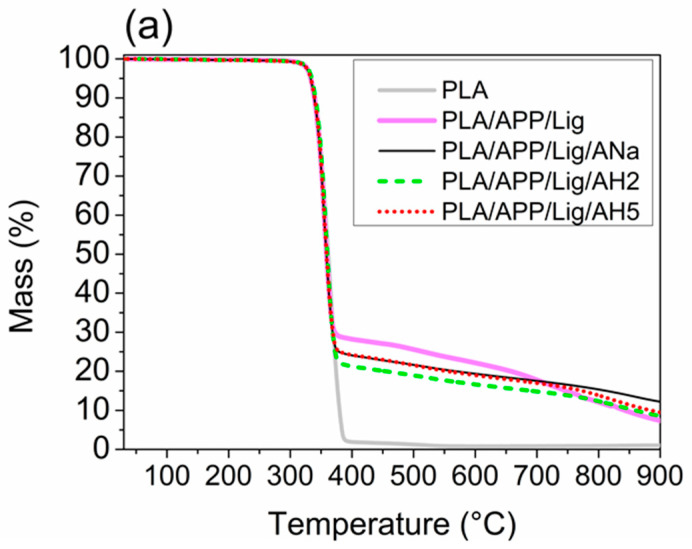

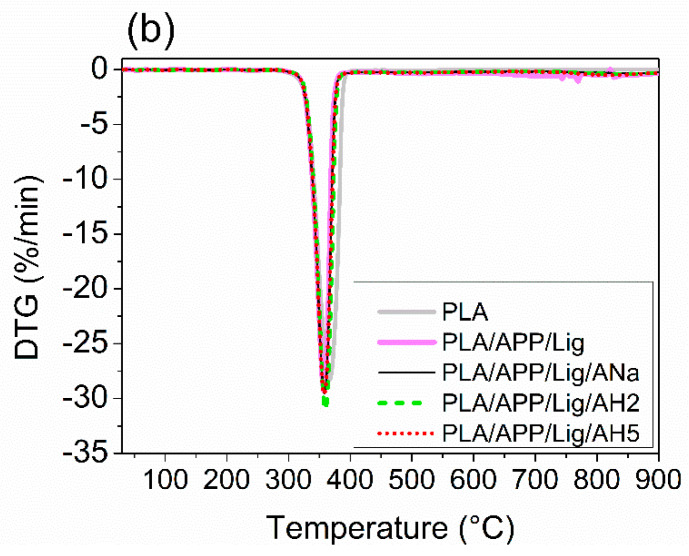
(**a**) Mass loss curves and (**b**) DTG curves for PLA and the composites.

Figure 1a,b present the experimental TGA and the derivative thermogravimetry (DTG) curves for the extruded pellets of PLA and the composites. All the mass loss curves (Figure 1a) are overlapped along the main degradation step until around 370 °C with similar values for the onset temperature (T_onset_) and for the temperature at DTG peaks (T_DTG peak_), as shown in Table 3. This step is associated to the degradation of the polymeric matrix as well as to the beginning of the lignin degradation and the release of ammonia [44]. The neat PLA presents a single degradation step, and it is completely consumed up at 550 °C, whereas the composites containing the intumescent formulation present more than one degradation step, with more thermally stable products being produced at 370 °C, promoting a significant decrease of the mass loss ratio when compared to the neat PLA. Despite of the very similar profiles of the composites’ curves, different percentages of the remaining residue are obtained for each one. The sole addition of ammonium polyphosphate and lignin to PLA is responsible for rising the final experimental residue from 0 to 7.4% in PLA/APP/Lig (see Table 3). This increase in residue can be attributed to the thermal decomposition of ammonium polyphosphate, which produces phosphoric acid and catalyzes the crosslinking between APP and lignin, with the formation of thermally stable products [53].

The addition of ANa and AH5 contributes to an increase of 65% in the final residue for the composite with ANa and of 28% for the one with AH5 when compared to PLA/APP/Lig. This increase in the final residue could be related to the enhancement of the thermal stability, which might be attributed to the interaction between the ammonium polyphosphate and the clay which can lead to the formation of aluminophosphate species [22,23]. Moreover, the mass difference of 1.2% between the final residues of PLA/APP/Lig/AH2 and PLA/APP/Lig, corresponds to the amount of AH2 added to the composite (see Section 2.3.1). Therefore, it seems that the time of the acidification has an important influence on the final residue of the samples. The AH5 and AH2 clays have a similar distribution of moderate strength sites, but the presence of strong Lewis acidic sites in AH2 can slow down the kinetics of the esterification reactions, giving rise to a less thermally stable char. On the contrary, the absence of those sites in ANa and in AH5 is an advantage, in addition to the presence of moderate strength Brønsted sites, resulting in the enhancement of the thermal stability of the condensed phase.

**Figure 2:**
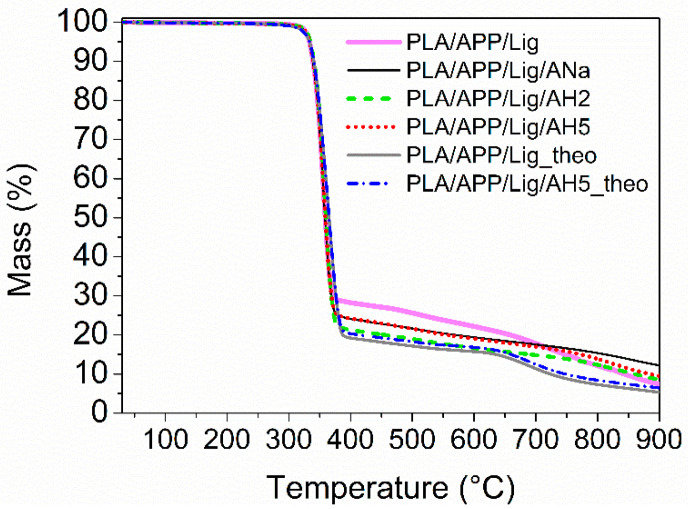
Theoretical and experimental mass loss curves for the composites.

The experimental and theoretical TGA curves for each system are compared in Figure 2 in order to verify the interaction between the composites additives. As the theoretical curves for PLA/APP/Lig/ANa, PLA/APP/Lig/AH2, and PLA/APP/Lig/AH5 are identical (i.e., their curves are superimposable as they present the same profile and the same amount of final residue), only the one for AH5 was chosen for comparison in Figure 2. The theoretical curves for PLA/APP/Lig and PLA/APP/Lig/clay present identical profiles, differing only in the amount of final residue at 900 °C (5.3% for PLA/APP/Lig and 6.4% for PLA/APP/Lig/clay, see Table 3). All the experimental curves show a larger percentage of final residue than the theoretical ones, revealing that the better thermal stability of the composites is a result of positive interactions between the components, and not only of a sum of individual effects. This interaction becomes more evident between 650 and 900 °C, a region where the theoretical curves show a more pronounced reduction of the thermal stability. Consequently, the experimental curves reach a higher percentage of final residue when compared to the predicted theoretically. For the composite without clay, PLA/APP/Lig, the final experimental residue is 40% higher than the predicted one. For the composites with clay, the final experimental residues are higher than the predicted ones by 34%, 48%, and 91% for PLA/APP/AH2, PLA/APP/Lig/AH5, and for PLA/APP/Lig/ANa, respectively.

#### Gas Phase Analysis of the Volatile Thermal Degradation Products through FTIR

**Figure 3:**
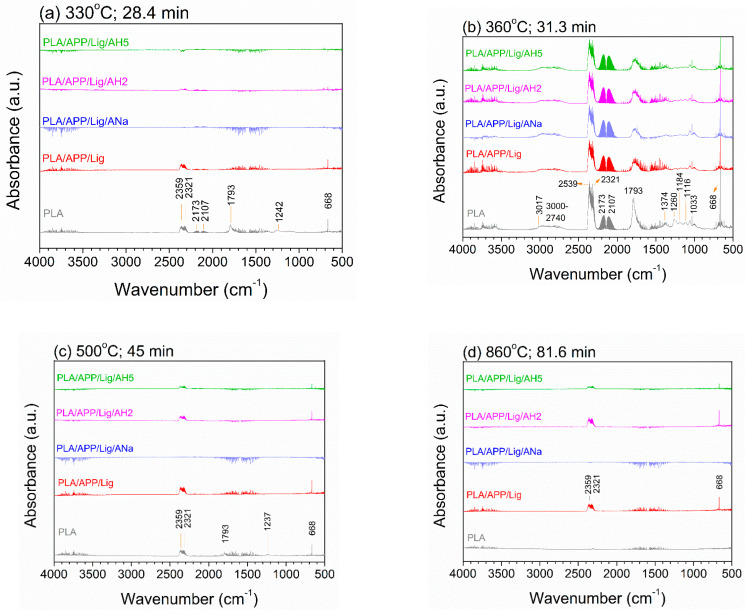
Gas phase TGA-FTIR spectra of PLA and the composites at (**a**) 330 °C; (**b**) 360 °C; (**c**) 500 °C; (**d**) 860 °C under air atmosphere.

Figure 3a–d displays the absorption spectra of the volatile species released during the degradation of the neat PLA and of the composites at different temperatures along the degradation process. The temperatures have been selected according to the TGA and DTG data (Table 3): 330 °C (close to T_onset_), 360 °C (close to T_DTG peaks_), 500 °C (along the thermal stabilization of the composites curves), and 860 °C (close to the end of the TGA test).

The PLA degradation process consists of a combination of a hydrolytic degradation (by cleavage of the esters groups, producing oligomers and monomers) and a thermal degradation (by chain scission and inter or intra trans-esterification reactions) [54]. The hydrolytic degradation is strongly influenced by the temperature, which tends to accelerate it, and by the acidity. In an acidic medium, the hydrolysis of the ester bonds is catalyzed by protons, producing low molecular weight oligomers, which are water soluble [54]. Table 4 summarizes the assignments of the absorption bands. At 330 °C (T_onset_, Figure 3a), the PLA degradation produces CO_2_ (2359, 2321, and 668 cm^−1^) and CO (2173 and 2107 cm^−1^). The band at 1793 cm^−1^ is associated to the gas phase carbonyl band of lactic acid [55], which is an expected product from the PLA hydrolysis [54,56]. The bands present in the 1300–1000 cm^−1^ range can be associated to the C-O stretching vibrations in esters and alcohols. At that temperature, only the composite without clay (PLA/APP/Lig) presents some significant bands (of CO_2_) besides the ones of PLA, indicating that the presence of clay slows down the release of volatile degradation products up to 330 °C.

Close to T_DTG peaks_ (360 °C, Figure 3b), the concentration of the degradation products of PLA increases, which can be inferred from the higher intensity of the corresponding bands. Furthermore, other volatile species are produced, as evidenced by new bands that can be assigned to alkene C-H stretching vibrations (3017 cm^−1^), alkane C-H stretching vibrations (3000–2740 cm^−1^), C-O bond of methanol (1033, 1000, and 1060 cm^−1^), and CH_3_ bending (1374 cm^−1^). The respective bands in all the composites show practically the same intensities but always lower than the respective ones in the neat PLA. The presence of the same volatile products in the neat PLA and in the composites evidences that they all have experienced the same thermal degradation process, which mainly consists of the breaking of the PLA ester bonds, leading to the formation of alcohol and carbonyl species. The lower intensity of the bands observed for the composites reveals that the intumescent formulation plays an important role in retarding the degradation process.

At 500 °C (Figure 3c), the decrease in the intensity of the bands and the disappearance of a great number of them are remarkable. For the neat PLA these effects are clearly due to the low amount of remaining polymer, almost completely consumed at this temperature. For the composites, this reduction can be attributed to the formation of thermally stable products (char), as they still conserve a high mass percentage at this temperature (see Figure 1a). PLA/APP/Lig, which does not contain any clay, presents the highest CO_2_ band intensity, followed by PLA/APP/Lig/AH2 and PLA/Lig/AH5, in a descending order of intensity. The PLA/APP/Lig/ANa composite is the only one that does not present any bands above its spectrum baseline, implying a more thermally stable product. This behavior is observed up to the end of the analysis at 860 °C (Figure 3d). On the other hand, PLA, already completely consumed, shows no bands, whereas PLA/APP/Lig and PLA/APP/Lig/AH2 present equivalent spectra in the region of the CO_2_ bands. Around this temperature the TGA curves of these two composites are practically overlapped, indicating a similar mass percentage, as shown in Figure 1a, while the PLA/APP/Lig/AH5 composite presents a spectrum with the lowest band intensities and also a high mass percentage. In contrast, the bands are completely absent in the PLA/APP/Lig/ANa composite which also exhibits the highest mass percentage at that temperature. These results corroborate the values of the final residues found for each system, indicating that a higher percentage of final residue is achieved by the more thermally stable char (Table 3), i.e., the char with the stronger ability to avoid the exchange of volatile products at higher temperatures.

Some works on intumescent composites report the participation of the polymeric matrix in the formation of the char [48,57,58]. A polypropylene (PP)/ammonium polyphosphate/polyamide-6 (PA-6) blend was prepared, containing either ethylene vinyl acetate (EVA) or ethylene–butyl acrylate–maleic anhydride (EBuAMA) as an interfacial agent [58]. The authors observed that the composite containing EVA showed a better fire performance than the one with EBuAMA, and suggested that the difference in performance could be related to an increase in the acidity of the medium caused by the functionalized co-monomers of EVA. The carboxylic species, originated from the thermal degradation of the EVA acetate groups, would take part in the condensed phase process. An analogous reasoning could be used to suggest that the carboxylate products of the thermal degradation of PLA could contribute to the char formation as an extra acid source. In spite of the fact that the acidity accelerates the thermal degradation of the PLA matrix, the formation of the carboxylate products could be an advantage due to the release of Brønsted acid species in the medium, promoting more efficient esterification reactions.

### 3.2. Cone Calorimetry (CC)

The values of the more relevant parameters obtained in cone calorimeter test, such as the time to ignition (TTI), the peak of heat release ratio (pHRR), the total heat released (THR), and the mass ratio of the remaining residue (M_residue_) are presented in Table 5. The% M_residue_ was calculated according to Equation (1):

(1) $${\%\;M}_{\mathrm{residue}}=\frac{M_{f}\left(g\right)}{M_{i}\left(g\right)}\times 100$$

where M_i_ and M_f_ stand for the initial and final mass of the sample.

**Figure 4:**
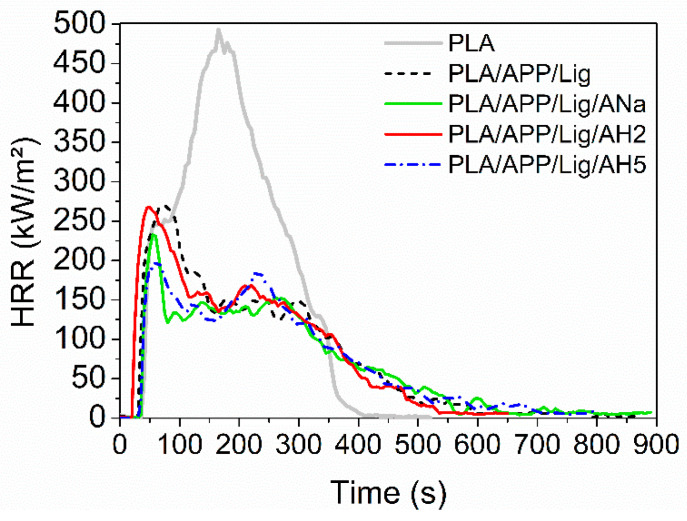
Heat release rate (HRR) curves for PLA and the composites submitted to injection molding.

Figure 4 presents the heat release rate (HRR) curves for PLA and the composites produced by injection molding. The effects of the addition of ammonium polyphosphate and lignin on the HRR curve profile of the neat PLA can be clearly perceived, as it is the only one showing a single degradation step. Besides, there is a dramatic decrease of the neat PLA pHRR value (498 kW m^−2^) when compared to the ones for all the composites. For example, relative to the PLA/APP/Lig composite, this reduction amounts to 48% (259 kW m^−2^), as shown in Table 5. In addition, for this same composite the THR was reduced by 40% relative to that of the neat PLA value. Overall, considering the increase of the remaining residue from 0 to 25% and the decrease of the heat released, the sole addition of the intumescent formulation was able to enhance the fire properties of the composite due to the formation of a protective intumescent layer (char).

The pHRR values for the PLA/APP/ANa and the PLA/APP/AH5 composites are lower than for the PLA/APP/Lig one, indicating that the addition of both clays has a greater positive impact on the flame-retarding properties than just APP and lignin. The addition of AH2 did not show any improvements when compared to the other clays, as its pHRR values remained very close to the ones of the composite without clay, PLA/APP/Lig. Amidst the clays, AH2 is the only one containing strong acidic sites, with a high excess of Lewis sites (Lpy) compared to the Brønsted (Bpy) ones (3 Lpy: 1 Bpy at 350 °C). Despite having a distribution of moderate strength sites similar to that of AH5, the presence of those strong sites represents a disadvantage for the esterification reactions. A possible explanation is that the coordination of the Lewis sites is more affected by steric hindrance, decreasing the esterification kinetics rate, as suggested in [33].

In conclusion, the composites containing ANa and AH5 presented the best performance in the cone calorimeter tests, with the decrease of the pHRR values, maintaining high values for the final residue. Differently from AH2, the ANa and AH5 clays do not contain strong acidic sites, only weak and moderate strength ones, a factor that can be associated to their better performance in comparison to the AH2 clay.

**Figure 5:**
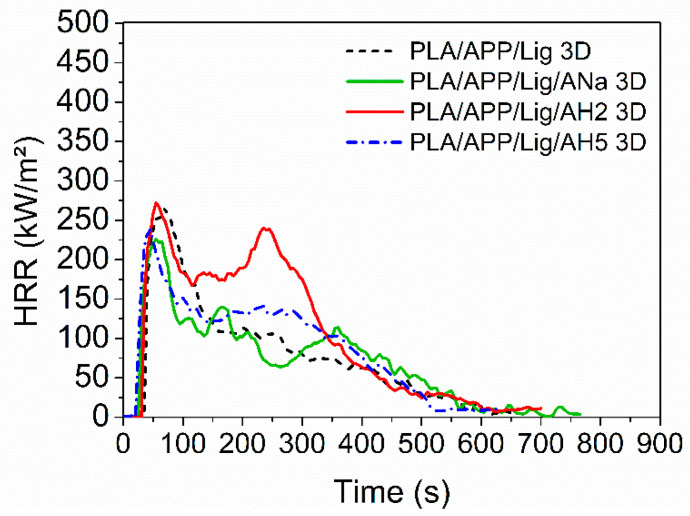
Heat release rate (HRR) curves for the composites submitted to additive manufacturing.

Although it was not possible to produce 3D-printed specimens of neat PLA (Ingeo™ Biopolymer 2003D) due to the lack of flexibility of the filament, the addition of the intumescent formulation made it possible to easily print the PLA composites. The HRR curves for PLA and the composites produced by FFF present similar profiles, except for the one containing AH2, which shows an intense second peak around 250 s, absent in all the other composites (Figure 5). Once again, the addition of AH2 clay did not cause any improvement in the pHRR when compared to those of the composite without clay, PLA/APP/Lig, despite presenting the lowest remaining residue among all the composites (Table 5). The pHRR values for the composites containing ANa and AH5 tend to be lower than the one for the PLA/APP/Lig 3D, but a decrease in the TTI values was only observed for PLA/APP/Lig/AH5 3D sample.

Differently from the samples submitted to injection molding, the 3D-printed ones containing clay presented a lower amount of mass residue when compared to the sample without clay, PLA/APP/Lig 3D. Overall, considering all the parameters obtained in the cone calorimeter tests, the PLA/APP/Lig/AH5 3D composite presented the best performance, followed by PLA/APP/Lig/ANa. The addition of AH2 was not effective to improve the fire-retardant properties. The performance of the 3D-printed composites with clay followed the same tendency observed for the injected ones: the presence of strong acidic sites, especially of Lewis’ ones, impairs the fire behavior, as in AH2. The absence of these acidic sites is responsible for a better performance in the CC tests.

Figure S1a–d shows the comparison between the HRR curves for each pair of injected and 3D-printed samples. Figure S1a,b,d exhibit the same change in their profiles, with a reduction of the heat release rate after the HRR peak. For the composites containing AH2 (Figure S1c) the HRR curve of the 3D-printed sample presents a more intense second peak (associated to the degradation of the char layer), around 250 s. This behavior might be related to the formation of a more fragile and less efficient char for the 3D-printed sample, unable to retain the volatile combustible products. The porosity created during the FFF printing and the consequent reduction of the sample density could be the reason for this fragility [7], promoting the exchange of oxygen and combustible volatiles between the internal and external parts of the char.

In general, the values of pHRR and THR for the injected and 3D-printed samples are equivalent. This feature was also observed in [6] using intumescent PLA nanocomposites with melamine polyphosphate and Cloisite 30B produced by FFF and hot press molding, as well as in [7] when evaluating the fire behavior of different formulations of flame-retarded PLA based on ammonium polyphosphate, melamine cyanurate, and Cloisite 30B or sepiolite.

### 3.3. FTIR Spectra of the Cone Calorimeter Residues

**Figure 6:**
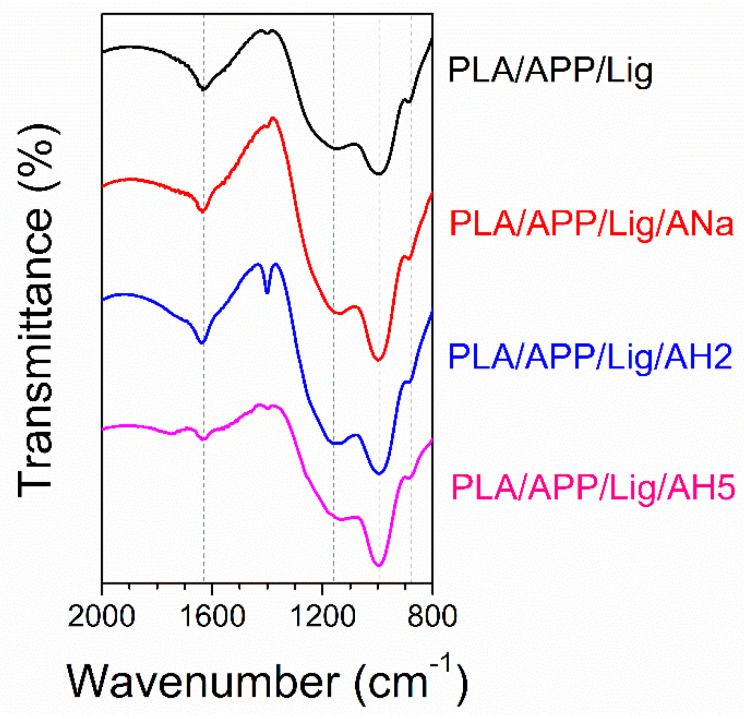
FTIR spectra of the composites char residues.

Table 6 summarizes the main bands in the FTIR spectra (Figure 6) of the injected composites char obtained after the CC tests. The phosphor-carbonaceous structures and the phosphate species exhibit bands in the range of 850–1350 cm^−1^ [64]. These bands are present in the FTIR spectrum of all the residues, indicating the formation of such structures. These bands correspond to the asymmetric vibration of the P-O bond in a P-O-P chain (886 cm^−1^), the symmetrical axial deformation of PO_2_ and PO_3_ in complex carbon phosphates (997 cm^−1^) and the stretching mode of P-O-C bonds in phosphate-carbon complexes (1128–1167 cm^−1^). The formation of P-O-C bonds and unsaturated compounds (evidenced by the C=C stretching vibrations at 1635 cm^−1^) are characteristic of the formed char bands. Only the residue of the PLA/APP/Lig/AH2 composite presents a significant chemical difference in comparison to the other char residues, with a remarkable narrow band at 1401 cm^−1^ assigned to the CH_2_ bending absorption. It is possible to associate the presence of this band to the poor flammability results obtained for PLA/APP/Lig/AH2 relative to the other clay-containing composites. The presence of these –CH_2_ groups could imply in a less efficient cyclization process due to the strong Lewis acidic sites present in AH2, which inhibits the esterification kinetics.

### 3.4. UL-94 and Limit Oxygen Index (LOI)

The pristine PLA did not achieve the minimum classification required by the UL-94 test. For both injected and 3D-printed samples, the addition of either the intumescent formulation or the intumescent formulation containing clay improved the performance, the composites being able to rapidly extinguish the flame in less than 50 s (t_1_ or t_2_ for each individual specimen <10 s and [t_1_ + t_2_] < 50s for the five specimens, a condition required for V-0 classification). However, all the samples promoted the ignition of the underneath cotton due to flammable dripping, denoting a V-2 classification. As the UL-94 test configuration consists on a specimen hanged up vertically, heated by a flame on its bottom surface, the dripping observed during the tests can be associated to the increase of the melt flow rate (MFR) caused by the addition of APP/Lig and clays (Table 7). PLA fabrics lignin/APP formulations were studied and it was observed that the incorporation of APP increases the MFR value due to the degradation of the PLA macromolecular chains promoted by the release of ammonia and phosphoric acid [44]. It was observed that the addition of lignin has the opposite effect, and the decrease of the MFR was attributed to the presence of hydroxyl groups in the lignin, enabling the formation of hydrogen bonds with the PLA matrix, consequently promoting more entanglement. They concluded that the addition of APP modifies the interactions PLA-lignin in PLA-APP and APP-lignin systems, resulting in the increase of the fluidity.

**Figure 7:**
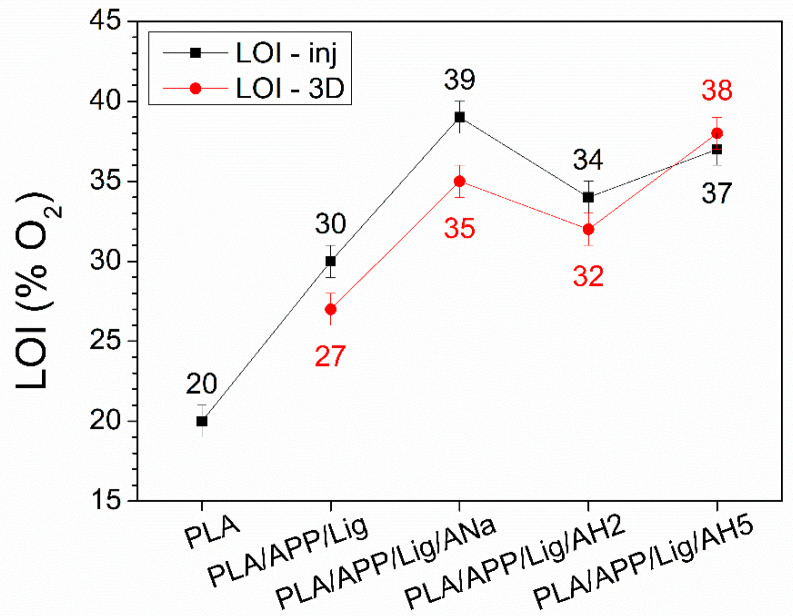
Comparison between LOI values for injected (inj) and 3D-printed (3D) samples.

The LOI results for the samples submitted to injection molding and to additive manufacturing are presented in Figure 7. The analysis of data for the injected samples clearly indicates that the addition of the intumescent formulation improves the fire-retarding properties of all the tested composites, increasing the LOI values to 39%, 37%, and 34% for the ANa, the AH5, and the AH2 composites, respectively.

Despite the 3D-printed samples present lower LOI values in comparison to the injected samples—probably due to the lower sample density related to the porosity induced by the FFF printing—a similar tendency is observed for the 3D-printed and injected composites. The LOI values are significantly higher for the clay-containing samples, 38%, 35%, and 32% respectively, for AH5, Ana, and AH2. The results obtained for these composites are higher than for some similar systems. A LOI value of 28.5% was found for both molded and 3D-printed nanocomposites made of 82% PLA, 17% melamine polyphosphate, and 1% Cloisite 30B [6].

For both processing methods (injection and FFF), the lowest LOI value is achieved for the composite containing AH2, the only clay with strong acidic sites, with a high excess of Lewis sites relative to the Brønsted ones. On the other hand, the highest LOI values are obtained for the composites with the ANa and AH5 clays, which do not contain strong acidic sites, but a more homogeneous distribution of Brønsted and Lewis with moderate strength.

A previous work [29] using these clays, poly(propylene) (PP) as the matrix, and ammonium polyphosphate and pentaerythritol as the intumescent formulation showed that moderate-strength sites were preferable to the strong ones, and that a better performance was achieved with an excess of Brønsted sites relative to the Lewis ones, highlighting the composite containing the AH5. Differently from PP, the degradation of PLA can produce protonated species which can act as an extra source of Brønsted acidity, contributing to the esterification reactions that give rise to char formation. The participation of the polymeric matrix in the char formation, already reported in [48,57,58], could explain the similar behavior of the composites with ANa and AH5 in the flammability tests, despite the higher ratio of Brønsted/Lewis sites of moderate strength in AH5.

### 3.5. Scanning Electron Microscopy (SEM) of the Injected Composites

**Figure 8:**
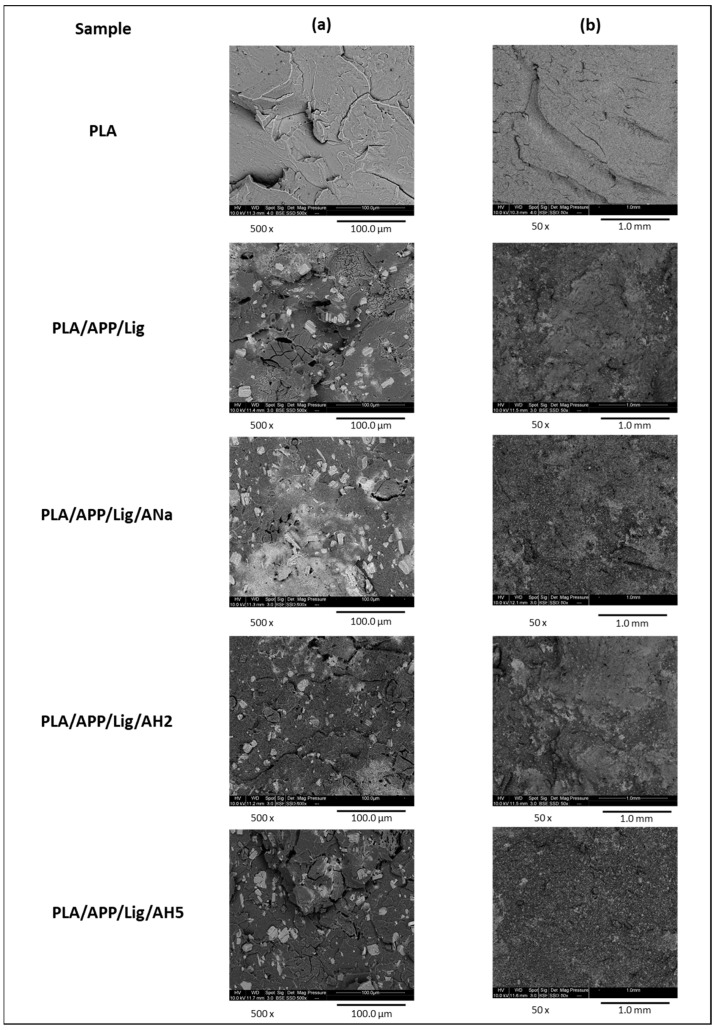
SEM images of the inner surface of PLA and the composites at (**a**) 500× and (**b**) 50× magnification.

Figure 8 shows SEM images of the inner surface of PLA and of the composites injected samples. At higher magnification (Figure 8, left column), it is possible to observe that there is no distinguishable difference in the distribution of the ammonium polyphosphate (white and cylindrical ones) and lignin (dark and spherical ones) particles, whereas the neat PLA has a homogeneous surface. At lower magnification (Figure 8, right column), the PLA surface is flat, while the surfaces of the composites are irregular and rugged, with similar characteristics of homogeneous particles distribution. These results eliminate the particles distribution as a possible source of the differences observed in the flammability tests of the composites.

### 3.6. Tensile Properties

**Figure 9:**
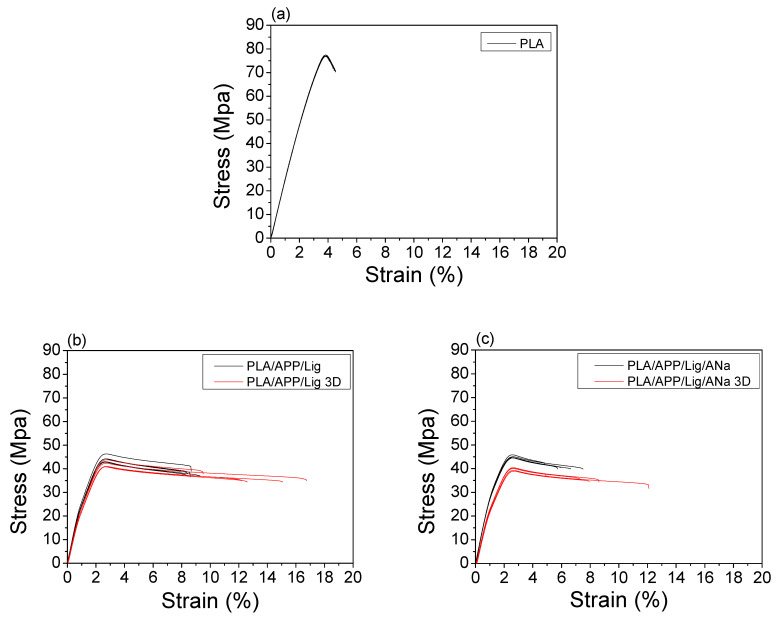

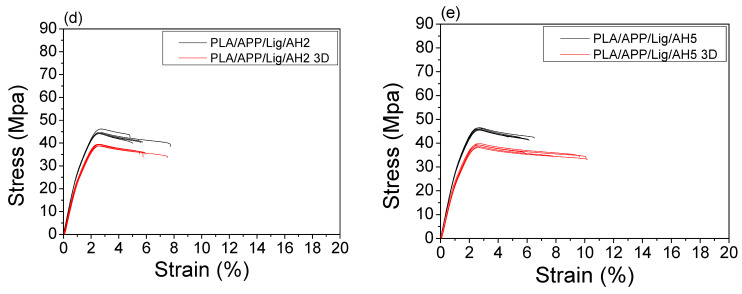
Stress X strain curves for (**a**) PLA; and comparison between injected and 3D-printed samples for (**b**) PLA/AP/Lig; (**c**) PLA/AP/Lig/ANa; (**d**) PLA/AP/Lig/AH2; (**e**) PLA/AP/Lig/AH5 for five specimens.

The main parameters extracted from the stress versus strain curves (Figure 9a–e) are reported in Table 8 for the injected and 3D-printed samples. The difference between the neat PLA and the injected composites is remarkable. PLA presents the highest yield strength and strength at break values but, at the same time, the lowest elongation at break and modulus values, making it more fragile and less rigid than the injected composites. The addition of the intumescent formulation regardless the presence of clay increased the ductility for all the composites, as evidenced by the higher values of the elongation at break, and the rigidity for the samples containing AH2 and AH5, as shown by the Young modulus values. Among the composites, a higher ductility was observed for the PLA/APP/Lig one (due to its higher elongation at break value) in comparison to the composites with clay. Hence, it can be noticed that the presence of the biobased component (lignin) tends to play a slight plasticizing effect on PLA.

There are no significant differences for the other parameters among the composites. The 3D-printed composites present stress versus strain curve profiles similar to those of the injected samples (Figure 9a–e) and no significant differences in their tensile parameter values (Table 8) were found.

**Figure 10:**
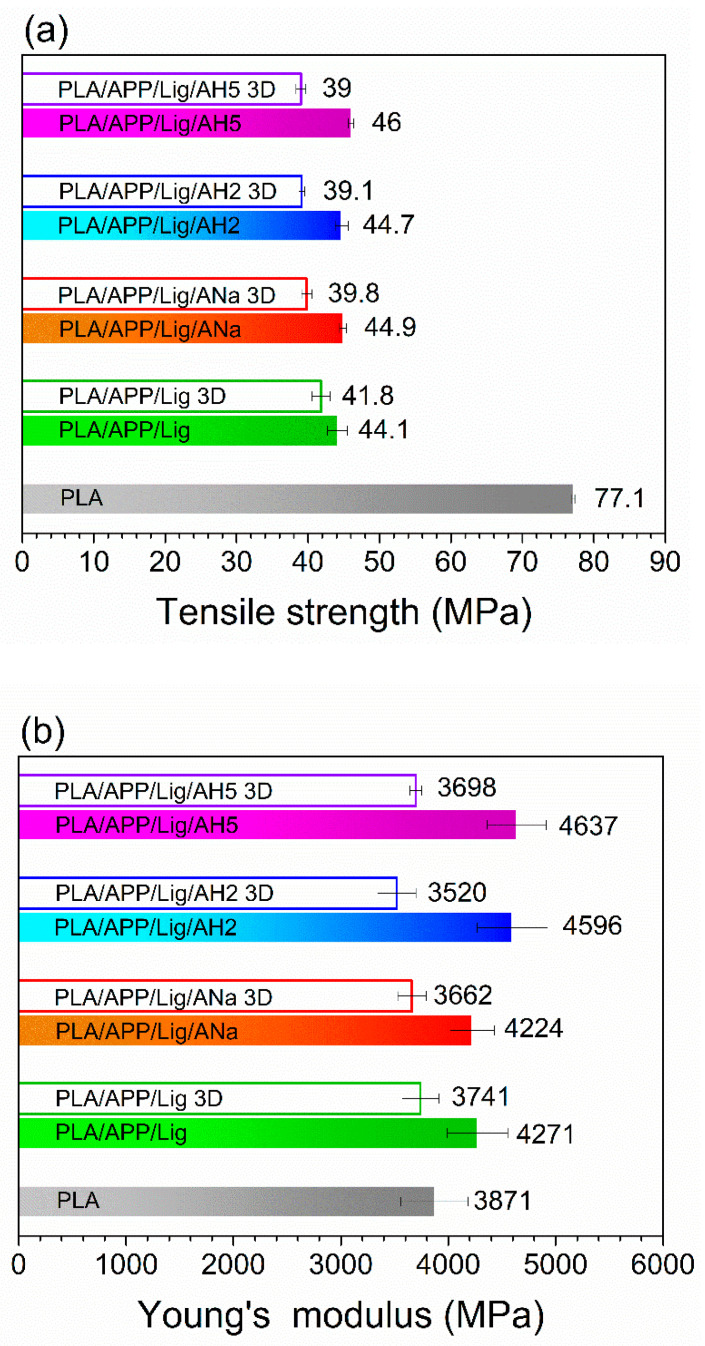
Comparison between injected and 3D-printed samples for (**a**) tensile strength and (**b**) Young’s modulus.

A comparison between the tensile strengths and the Young modulus for the injected and 3D-printed samples is shown in Figure 10a,b. The sole addition of the intumescent formulation does not promote a significant difference in the tensile strength values of the injected and 3D-printed samples (Figure 10a), but with the addition of clay, the injected composites show higher tensile strength values than the 3D-printed ones. This difference can be explained by the formation of voids during the layers deposition in the additive manufacturing, leading to a limited adhesion between these layers and producing crack points and entailing the weakening of the material, consequently decreasing the tensile strength [2]. PLA composites with melamine polyphosphate and Cloisite 30B, prepared by hot press molding and FFF, also present lower values for the samples submitted to the additive manufacturing (70.0 ± 3.8 MPa) than for the molded ones (80.4 ± 4 MPa) [20].

For the Young’s modulus (Figure 10b), all the injected composites show higher values than the 3D-printed ones, indicating, in this case, that the injection is able to produce more rigid materials than the additive manufacturing, probably due to the limited adhesion between the filaments in the 3D process. The possible contribution to tensile properties due to a supposed higher orientation for the macromolecules processed through 3D-printing seems offset by the presence of voids and the lack of adhesion between the PLA layers.

## 4. Conclusions

This work studied the influence of Brønsted and Lewis acidity of acidic-activated montmorillonites on the flame-retardancy and mechanical properties of intumescent PLA composites produced by both injection molding and FFF techniques. The intumescent formulations were composed of ammonium polyphosphate, lignin, raw and acidic-activated montmorillonites.

In general, compared to the neat PLA, the addition of the intumescent formulation and clays increased the ductility and the rigidity of the composites, both for the injected and the 3D-printed samples. The injected samples present higher Young modulus than those of the 3D-printed ones. The tensile strength values are also higher for the injected composites when compared to the 3D-printed ones, except for the PLA/APP/Lig one, which showed similar values for both the injected and the 3D-printed samples. It can be suggested that the lack of adhesion and void formation is favored by the presence of clays.

Neat PLA did not classify in UL-94 test, while all the injected and 3D-printed composites achieved a V-2 classification. For both processing methods (injection and FFF), the LOI tests indicate a significant enhancement of the fire behavior with the addition of the intumescent formulation APP/Lig. An even better performance was reached by the composites containing clay, the highest LOI values being achieved by the PLA/APP/Lig/ANa and PLA/APP/Lig/AH5 composites.

The thermogravimetry and flammability results evidence that the strong acidic sites composed mostly by the Lewis type are detrimental for the thermal stability and the fire-retarding properties. Conversely, the absence of these sites and the presence of moderate strength ones enhance the fire behavior. A similar behavior was observed both in cone calorimeter and LOI tests for the clay-containing samples submitted to injection and FFF, showing that the influence of the montmorillonite acidity is independent of the processing technique. The samples containing AH2, with strong Lewis sites, presented the worst performance, while the ones with ANa and AH5 (which do not contain strong acidic sites, but a more homogeneous distribution of Brønsted and Lewis with moderate strength sites) achieved the best results. The strong sites present in AH2, mostly of the Lewis type, can compromise the esterification kinetics and, consequently, impair the cyclization reactions, as evidenced by the presence of a remarkable narrow band at 1401 cm-1 (CH2 bending absorption) in the FTIR spectrum of the cone calorimeter residue.

Overall, this work brings out a new insight on the influence of montmorillonite on a novel bio-based intumescent composite, promoting a deeper comprehension on how the chemistry of the acidic sites of the montmorillonite can help to tune the flammability properties of intumescence composites leading to the development of more efficient flame-retarding materials.

## Figures and Tables

**Table 1:** Concentration of Brønsted (Bpy) and Lewis (Lpy) acidic sites, measured by FTIR-Pyr [29].

Sample	Concentration of Acidic Sites					
	150 °C (Weak Sites)		250 °C (Moderate Strength Sites)		350 °C (Strong Sites)	
	Bpy (%)	Lpy (%)	Bpy (%)	Lpy (%)	Bpy (%)	Lpy (%)
ANa	50	50	31	69	-	-
AH2	52	48	51	49	25	75
AH5	48	52	55	45	-	-

**Table 2:** Equations used to construct the TGA theoretical curves for each composite.

Composite	*M*_theo_ (*T*)
PLA/APP/Lig	*M*_theo_ (*T*) = 0.80 *M*_PLA_ (*T*) + 0.20 *M*_IF_ (*T*)
PLA/APP/Lig/ANa	*M*_theo_ (*T*) = 0.788 *M*_PLA_ (*T*) + 0.20 *M*_IF_ (*T*) + 0.012 *M*_ANa_ (*T*)
PLA/APP/Lig/AH2	*M*_theo_ (*T*) = 0.788 *M*_PLA_ (*T*) + 0.20 *M*_IF_ (*T*) + 0.012 *M*_AH2_ (*T*)
PLA/APP/Lig/AH5	*M*_theo_ (*T*) = 0.788 *M*_PLA_ (*T*) + 0.20 *M*_IF_ (*T*) + 0.012 *M*_AH5_ (*T*)

*M*_theo_ (*T*): theoretical mass loss as function of the temperature, *T*. *M* (*T*): experimental mass loss as function of the temperature, *T*. IF: intumescent formulation, composed by the sum of the ratios of APP and Lig used for each composite, as presented in Section 2.3.1.

**Table 3:** Data for TGA and DTG curves for PLA and the composites produced.

Sample	T_onset_ (°C)	T_DTG peak_ (°C)	Experimental Residue at 900 °C (% Mass)	Theoretical Residue at 900 °C (% Mass)
PLA	324	366	0	-
PLA/APP/Lig	318	358	7.4	5.3
PLA/APP/Lig/ANa	324	366	12.2	6.4
PLA/APP/Lig/AH2	323	360	8.6	6.4
PLA/APP/Lig/AH5	325	357	9.5	6.4

**Table 4:** Assignments for the absorption bands for TGA-FTIR spectra of PLA and the composites.

Absorption Bands (cm^−1^)	Assignments	References
2359, 2321 and 668	CO_2_	[59,60,61]
2173 and 2107	CO	[60,61]
1793	C=O stretching vibration of lactic acid	[55]
1440–1220	C-O-H bending vibration in alcohols coupled to H-C-H bending vibration	[62]
1374	CH_3_ bending	[62]
1300–1000	C-O stretching vibrations in alcohols and esters	[62]
1033, 1000 and 1060	C-O bond of methanol	[63]

**Table 5:** Cone calorimeter results obtained for PLA and the composites submitted to injection molding and FFF.

Sample	TTI (s)	pHRR (kW m^−^²)	THR (MJ m^−^²)	M_residue_ (%)
PLA	36 ± 3	498 ± 6	95 ± 1	0
PLA/APP/Lig	31 ± 2	259 ± 11	57 ± 9	25 ± 6
PLA/APP/Lig/ANa	34 ± 4	229 ± 6	58 ± 1	31 ± 2
PLA/APP/Lig/AH2	27 ± 6	259 ± 20	60 ± 7	25 ± 4
PLA/APP/Lig/AH5	34 ± 2	213 ± 18	56 ± 8	30 ± 3
PLA/APP/Lig 3D	31 ± 8	251 ± 14	50 ± 2	38 ± 1
PLA/APP/Lig/ANa 3D	28 ± 3	225 ± 13	54 ± 3	36 ± 4
PLA/APP/Lig/AH2 3D	31 ± 7	268 ± 16	55 ± 6	21 ± 5
PLA/APP/Lig/AH5 3D	21 ± 0	226 ± 14	45 ± 5	33 ± 6

**Table 6:** Assignment of the bands for the FTIR spectra of the composites char residues.

Bands (cm^−1^)	Assignment	References
886	asymmetric vibration of the P-O bond in a P-O-P chain	[39,64,65]
997	symmetrical axial deformation of PO_2_ and PO_3_ in complex carbon phosphates	[64,66]
1128–1167	stretching mode of P-O-C bonds in phosphate-carbon complexes	[39,64,66]
1401	CH_2_ bending absorption	[39]
1635	C=C stretching vibrations	[39,61,67]

**Table 7:** Melt flow rate for the neat PLA and the composites.

Sample	MFR (g 10 min^−1^)
PLA	6
PLA/APP/Lig	15
PLA/APP/Lig/ANa	18
PLA/APP/Lig/AH2	20
PLA/APP/Lig/AH5	23

**Table 8:** Tensile test results for the injected and 3D-printed samples for five specimens per sample.

Sample	Yield Strength	Yield Strain	Strength at Break	Elongation at Break	Modulus
	(MPa)	(%)	(MPa)	(%)	(MPa)
PLA	77.1 ± 0.3	3.82 ± 0.02	72 ± 2	4.4 ± 0.2	3871 ± 311
PLA/APP/Lig	44.1 ± 1.4	2.66 ± 0.05	37 ± 1	8.7 ± 0.4	4271 ± 282
PLA/APP/Lig/Ana	44.9 ± 0.5	2.57 ± 0.05	40 ± 1	6.1 ± 1.0	4224 ± 205
PLA/APP/Lig/AH2	44.7 ± 0.9	2.59 ± 0.07	40 ± 1	5.8 ± 1.2	4596 ± 328
PLA/APP/Lig/AH5	46.0 ± 0.4	2.62 ± 0.04	42 ± 1	5.8 ± 0.7	4637 ± 273
PLA/APP/Lig 3D	41.3 ± 1.3	2.70 ± 0.03	35 ± 1	13.2 ± 2.8	3741 ± 170
PLA/APP/Lig/ANa 3D	39.8 ± 0.7	2.62 ± 0.04	35 ± 2	8.2 ± 2.6	3662 ± 133
PLA/APP/Lig/AH2 3D	39.1 ± 0.4	2.57 ± 0.05	35 ± 1	6.1 ± 0.8	3520 ± 178
PLA/APP/Lig/AH5 3D	39.0 ± 0.7	2.63 ± 0.04	34 ± 1	8.7 ± 1.9	3697 ± 53

## Data Availability

Not applicable.

## Supplementary Materials

The following supporting information can be downloaded at: https://www.mdpi.com/article/10.3390/polym14091702/s1. Figure S1: Comparison of heat release rate curves of the injected and the 3D-printed samples.
