# Supplementary material for: Flame-Retarding Properties of Injected and 3D-Printed Intumescent Bio-Based PLA Composites: The Influence of Brønsted and Lewis Acidity of Montmorillonite
Source: Polymers (Basel). 2022 Apr 21;14(9):1702. doi: 10.3390/polym14091702 (PMC9105856; doi:10.3390/polym14091702)
Supplement: Supplementary file 1 [file polymers-14-01702-s001.zip › polymers-1650027-supplementary.pdf]

polymers

MDPI

Supplementary Materials: Flame-Retarding Properties of injected and 3D-Printed Intumescent bio-based PLA Composites: the Influence of Brønsted and Lewis Acidity of montmorillonite

Raíssa Carvalho Martins, Simone Pereira da Silva Ribeiro, Michelle Jakeline Cunha Rezende, Regina Sandra Veiga Nascimento, Marco Antonio Chaer Nascimento, Marcos Batistella and José-Marie Lopez-Cuesta

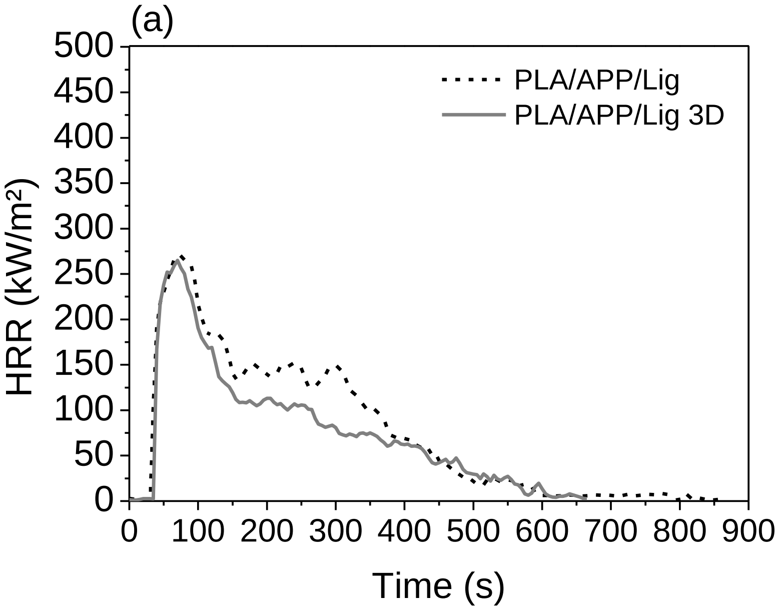

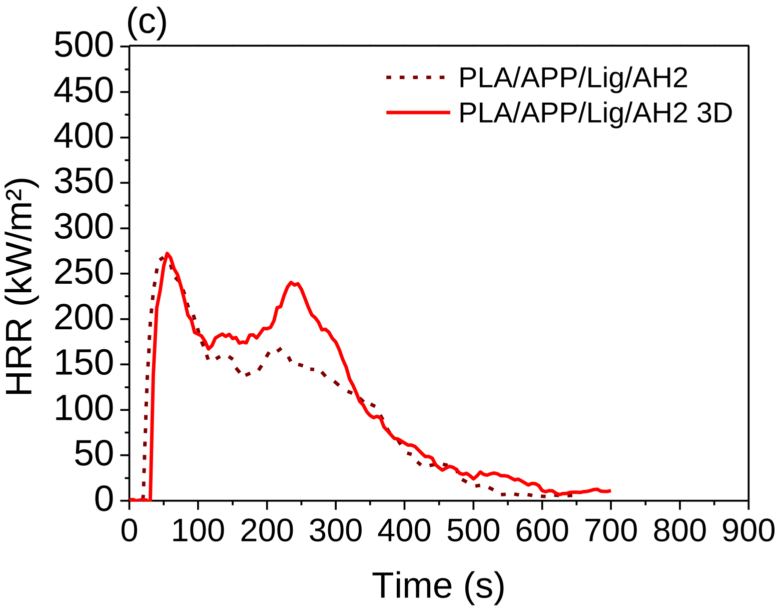

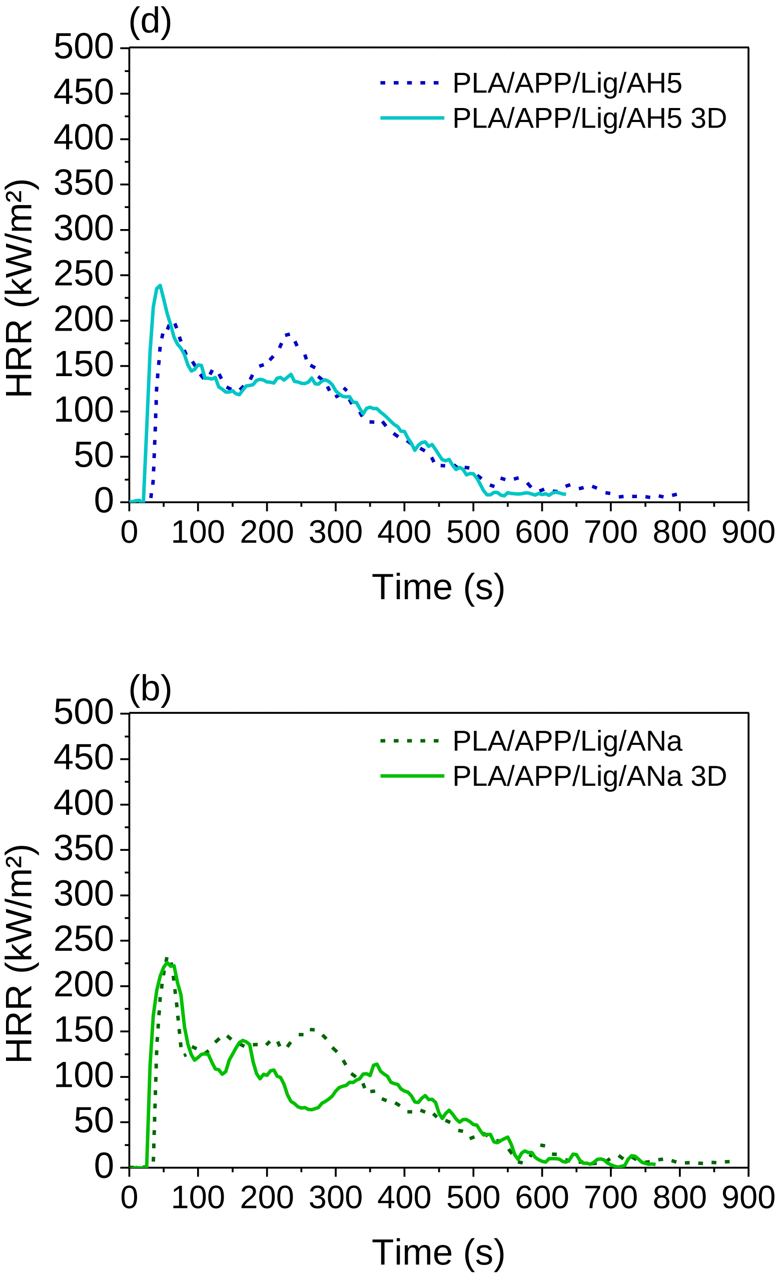

Figure S1. Comparison of heat release rate curves of the injected and the 3D-printed samples.
